# Assessing consciousness in patients with disorders of consciousness using soft-clustering

**DOI:** 10.1186/s40708-023-00197-5

**Published:** 2023-07-14

**Authors:** Sophie Adama, Martin Bogdan

**Affiliations:** Department of Neuromorphe Information Processing, Leipzig University, Augustusplatz 10, Leipzig, 04109 Germany

**Keywords:** Brain–computer interface, Complexity, Connectivity, Consciousness, Disorders of consciousness, Electroencephalogram, Soft-clustering, Spectral analysis

## Abstract

Consciousness is something we experience in our everyday life, more especially between the time we wake up in the morning and go to sleep at night, but also during the rapid eye movement (REM) sleep stage. Disorders of consciousness (DoC) are states in which a person’s consciousness is damaged, possibly after a traumatic brain injury. Completely locked-in syndrome (CLIS) patients, on the other hand, display covert states of consciousness. Although they appear unconscious, their cognitive functions are mostly intact. Only, they cannot externally display it due to their quadriplegia and inability to speak. Determining these patients’ states constitutes a challenging task. The ultimate goal of the approach presented in this paper is to assess these CLIS patients consciousness states. EEG data from DoC patients are used here first, under the assumption that if the proposed approach is able to accurately assess their consciousness states, it will assuredly do so on CLIS patients too. This method combines different sets of features consisting of spectral, complexity and connectivity measures in order to increase the probability of correctly estimating their consciousness levels. The obtained results showed that the proposed approach was able to correctly estimate several DoC patients’ consciousness levels. This estimation is intended as a step prior attempting to communicate with them, in order to maximise the efficiency of brain–computer interfaces (BCI)-based communication systems.

**Supplementary Information:**

The online version contains supplementary material available at 10.1186/s40708-023-00197-5.

## Introduction

Disorders of consciousness (DoC) states encompass the states in which an individual’s consciousness is impaired. Consciousness relies upon the interaction between the activity of the thalamus, the brainstem and the cerebral cortex of the brain. Damages in one of these systems (e.g., after a brain injury) will disrupt this relationship, and result in an impairment of consciousness which is called DoC [1]. One can distinguish coma, vegetative state (VS) formerly known as Unresponsive Wakefulness Syndrome (UWS), and Minimally Conscious State (MCS) [1]. Patients can be in coma for 2 to 4 weeks during which they are “unarousable”. This state is characterised by an absence of spontaneous eyes opening and muscle movements [2, 3]. If and when patients emerge from this state, they can enter either a locked-in or a vegetative state, which in turn can transition to an MCS, or in the worst-case scenario, into permanent VS and/or death.

To assess DoC patients’ consciousness, most researches rely on their active participation using event-related potentials in particular, since it proves the patients’ ability to follow commands, which in turn is seen as proof of consciousness [2]. The stimuli used in this case can be auditory, tactile, visual or even olfactory [4–8]. This easily provokes patients’ fatigue. Furthermore, most studies do not evaluate patients’ consciousness and willingness to perform the tasks. In this work, several features were extracted from the EEG signal and used as input to two clustering analysis approaches that subsequently issue an estimation of the consciousness level of the DoC patients. The idea behind this is to maximise the probability of correctly determining the patients’ actual states at each time, given that signature of probable consciousness detected by one feature can be missed by another.

This paper is organised as follows: the patients as well as the data recorded from them are presented in Sect. 2. This is followed by the introduction of the different methods used to extract the EEG features. Afterwards, the soft-clustering algorithms used to determine the patients’ levels of consciousness are described. Then the results are presented in Sect. 3 and discussed in Sect. 4, before concluding in Sect. 5.

## Methods

### Patients description

The dataset consists of the EEG recordings of two subgroups of patients: 11 in MCS and 12 in VS from Austria and Belgium [9]. The complete dataset was published in [10] and a brief description of them is given in Table 1.

**Table 1:** Demographic information of the patients

Patient	Age/gender	DoC	Aetiology	Duration^a^	CRS-R^b^
L1	21/M	VS	TBI^c^	7	6
L3	16/F	VS	TBI	1	7
L13	74/F	VS	TBI	1	3
S12	52/M	VS	TBI	13	4
S13	58/F	VS	CVA^d^	28	4
S14	61/M	VS	Anoxia^e^	32	4
S16	50/F	VS	CVA	45	4
S17	19/M	VS/MCS	SSPE^f^	24	3
L4	48/M	MCS	TBI	8	11
L7	66/M	MCS	CVA	3	10
L8	62/M	MCS	TBI	2	8
L9	61/M	MCS	Anoxia	2	10
L16	43/F	MCS	TBI	6	21
S2	45/M	MCS	TBI	12	8
S5	21/M	MCS	Anoxia	28	13
S6	50/F	MCS	TBI	113	14
S7	30/M	MCS	TBI	120	13

^a^Months since injury

^b^CRS-R: Coma Recovery Scale-Revised, measure to determine consciousness levels of unresponsive patients and establish a diagnosis

^c^TBI: traumatic brain injury

^d^CVA: cerebrovascular accident

^e^Anoxia: condition characterised by an absence of oxygen supply to an organ or a tissue

^f^SSPE: subacute sclerosing panencephalitis. SSPE is a progressive neurological disorder targeting children and young adults and affecting the central nervous system (CNS). It is a rare disease caused by a slow and persistent viral infection associated to measles [11]

**Fig. 1:**
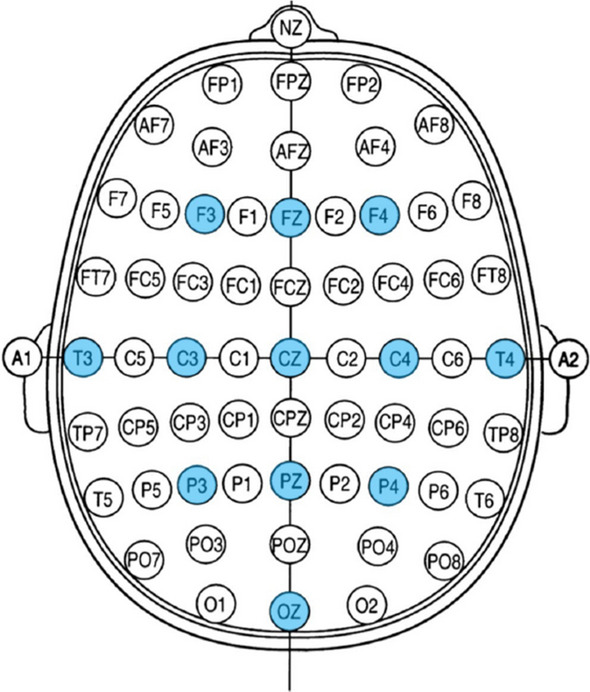
Illustration of the common EEG channels to all DoC patients (in blue). The sampling rate is 500 Hz. Patients were based in Austria (Austrian group) and in Belgium (Belgian group) [9]

The EEG were acquired from 18 channels placed according to the 10–20 system [12] for the Austrian group, and 12 channels for the Belgian group at a sampling rate of 500 Hz. In this work, the analysis was performed on the channels common to both groups, as illustrated in Fig. 1. In addition to the EEG, physiological signals were also recorded. Moreover, video recordings were also labelled into periods of “eyes open” (O) and “eyes closed” (C) for every 5-min epoch for the 23 patients. When the state of the eyes repeatedly switched between opening and closure, it was scored as open–closed (O/C) [10].

### Description of the approach

**Fig. 2:**
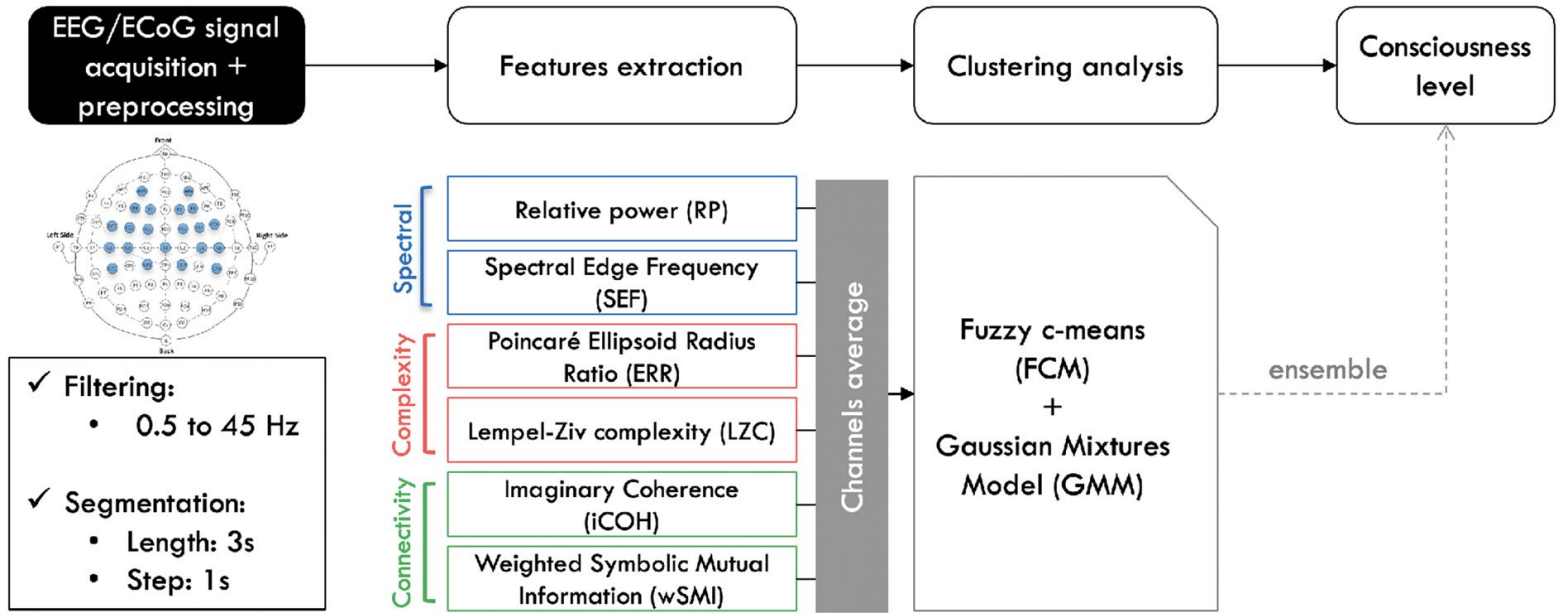
Signal processing and analysis pipeline. The recorded signal is filtered and segmented, before extracting the different features. Each feature is then averaged across selected group of channels before performing the clustering analysis. The probability that the patient is conscious is then extracted by applying a decision rule to the obtained results [14]

All analyses were performed using MATLAB 2018b, the FieldTrip toolbox [13], as well as custom written scripts. Figure 2 illustrates the modus operandi of the proposed method. The acquired EEG data were first band-pass filtered from 0.5 to 45 Hz using a third-order Butterworth filter, and then segmented into 3-s windows sliding one second at a time. No artefacts removal were performed on the data given the states of the patients. Afterwards, the features of interest are computed for each segment and for all channels, and subsequently averaged across them. Then, soft clustering analyses are performed on the extracted features to obtain a unique value estimating the patients’ consciousness levels.

It is hypothesised that conscious states are characterised by:

- A simultaneous increase of the $\theta$ and $\beta$ powers, since on one hand the former increase during verbal and spatial memory tasks [15] and on the other hand, the latter is highly activated during information processing in the brain [16, 17].
- Spectral edge frequencies 95% (SEF95) above the $\alpha$ band. In anaesthesia research, values below the $\alpha$ band indicate deep level [18] while those above the $\beta$ band characterise light anaesthesia [19].
- Larger EEG complexity, as complex signals are representative of an activated brain [20].
- Increased linear and non-linear connectivity in the $\theta$ band.

Therefore, for each of these different features, the clusters centroids with higher values are considered to be representative of conscious states.

#### Features computation

Different types of EEG signal characteristics were used in this study, namely frequency, complexity, and connectivity-based features. The relative powers (RPs) of $\theta$ and $\beta$, as well as the SEF95 were considered. As complexity measures, the Ellipsoid Radius Ratio (ERR) of the Poincaré plots [21] and the Lempel–Ziv complexity (LZC) [22] were used. Brain connectivity was determined using the imaginary part of the coherency (iCOH) [23], and weighted symbolic mutual information (wSMI) [24]. The details of each of these features are developed in the following paragraphs.

##### Spectral features

In normal circumstances, the values of the different frequency powers provide information about the (current) brain states [16]. For a signal *x*(*t*), the relative power is obtained using Eq. (1) [25, 26]. The frequency bands of interest are $\theta$ (4–8 Hz) and $\beta$ (12–30 Hz):

1 $$\begin{aligned} {\text{RP}} = \frac{\sum _{f=f_1}^{f_2} S_x(f)}{\sum _{f=f_l}^{f_h} S_x(f)}, \end{aligned}$$

where $S_x(f)$ is the power spectral density (PSD) of the signal *x*(*t*) at the frequency *f* [27], $f_1$ and $f_2$ specify the lower and upper limits of the frequency band of interest, respectively. In this particular case, $f_l = 0$ Hz and $f_h = 45$ Hz (i.e. the upper limit of the cut-off frequency of the filter). Practically, $S_x(f)$ was estimated using the MATLAB function SPSVERBc1 with a Hamming window of 1/8 size of the data segment and a 50% overlap, using the Welch method [28].

Investigations of the potential of the relative powers as markers of consciousness in patients with DoC showed that $\theta$ and $\alpha$ are among the best features that could distinguish MCS from VS patients. Furthermore, an increase of $\theta$ power is detected during verbal and spatial memory tasks [15] and throughout the recovery of consciousness after anaesthesia [29]. On the other hand, $\beta$ rhythms (13–30 Hz) are produced when the brain is engaged in information processing [25].

SEF represents the frequency beneath which a particular fraction *r* of the signal power is contained [30, 31] and is computed using Eq. (2), where *f* is the frequency and *Fs* represents the sampling frequency. In this research, normalisation of the values of SEF was performed by dividing them to the upper frequency limit of the critical frequency during filtering (45 Hz):

2 $$\begin{aligned} \sum _{f=0}^{\text{SEF}_r} S_x(f) = r \sum _{f = 0}^{Fs/2} S_x(f). \end{aligned}$$

SEF are generally used features for sleep analysis and classification, with $r=50\%$ and $r=95\%$. SEF95 (SEF with $r=95\%$) in particular is usually employed in anaesthesia research to evaluate the depth of anaesthesia in healthy subjects. Its value decreases as the anaesthesia level deepens [18]. More precisely, SEF95 values larger than 15 Hz indicate light anaesthesia. Moderate anaesthesia is characterised by SEF95 values between 8 and 13 Hz, while deep anaesthesia have SEF95 values lower than 7 Hz [19]. Accordingly, a bigger SEF95 value indicates a higher level of consciousness.

##### Complexity features

The complexity of EEG signals were assessed using the ERR of Poincaré plots and LZC. More complex signals are representative of more brain activation, hence higher consciousness states [21, 32, 33].

**Fig. 3:**
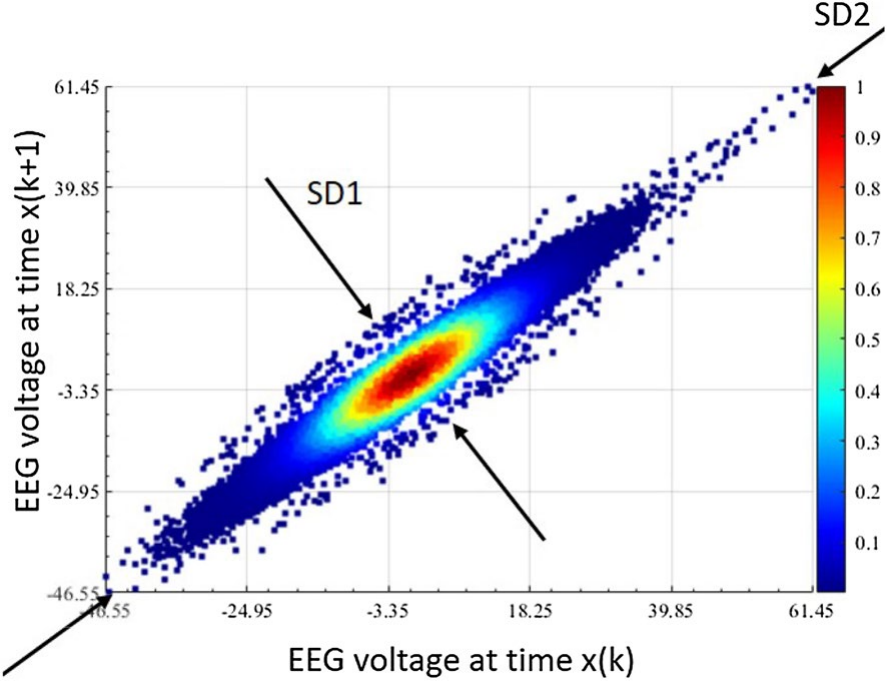
Poincaré plot showing its short-term (SD1) and long-term (SD2) variability with $\tau = 1$ sample. A round oval pattern of the plot represents a random signal, while an elongated shape describes signals with linear features

A Poincaré plot describes the behaviour of the signal in the phase space [21]. To obtain it, the signal *x*(*t*) is plotted against its delayed version $x(t+\tau )$. Figure 3 illustrates an example of Poincaré plot of EEG data with $\tau =1$ sample. SD2 and SD1 are, respectively, the standard deviation of the points from the long axis (line of identity) and the short axis (perpendicular to the line of identity) [34, 35]. The variable of interest is the ERR, which is the ratio SD1/SD2, and is calculated using Eq. (3):

3 $$\begin{aligned} {\text{ERR}} = \frac{{\text{SD}}1}{{\text{SD}}2} = \frac{\frac{\sqrt{2}}{2}{\text{SD}}(x(t)-x(t+\tau ))}{\sqrt{2{\text{SD}}(x(t))^2-\frac{1}{2}SD(x(t)-x(t+\tau ))^2}}. \end{aligned}$$

An increased depth of anaesthesia is characterised by a reduced randomness of the EEG signal and the short-term variability SD1 and, by extension, of the ERR [35]. A rounder shape of the ellipsoid (ERR $\approx$ 1) corresponds to randomness, thus more complex signals. Consequently, the closer to 1 the value is, the higher the consciousness level is.

On the other hand, LZC assesses repetitiveness in a binary sequence $S = s_1 s_2... s_n$ [22]. It determines the number of different sub-strings found as the binary sequence is streamed from the left to the right. Larger number of sub-sequences are representative of a higher degree of randomness, which increase the LZC [36, 37]. The binary sequence is obtained by transforming the real signal *x*(*t*) to an analytic signal using Eq. (4) and binarising it using Eq. (5):

4 $$\begin{aligned} x_a(t) = x(t)+\textit{i}x_h(t), \end{aligned}$$

where $x_h(t)$ is the Hilbert transform of *x*(*t*) [38].

5 $$\begin{aligned} S(t) = {\left\{ \begin{array}{ll} 0, &{} \text {if } {\text{abs}}\left( x_h(t) \right) \le {\text{mean}}\left( {\text{abs}}\left( x_h(t) \right) \right) \\ 1, &{} \text {otherwise} \end{array}\right. } \end{aligned}$$

A normalised version of LZC was recently used to assess consciousness levels of different types of patients compared to healthy controls [39].

##### Connectivity features

The different brain regions communicate with one another during mental tasks. Investigating this may shed some light on the underlying brain processes. Generally, high connectivity values indicate high cooperation and more information sharing between the two underlying brain regions or channels [40]. Two different measures, iCOH and wSMI, were used in this case.

Coherency assesses the linear relation between a pair of signals, channels or brain regions *x* and *y*. An increased functional relationship between these regions is reflected by a higher value of coherence [41]. To reduce the influence of volume conduction in the brain, only the imaginary part of the coherency is used [23]. Its value at a frequency *f* for each pair of channels is obtained using Eq. (6) and normally ranges from $-1$ to $+1$:

6 $$\begin{aligned} i{\text{COH}}_{xy}(f) = \Im \left( \frac{S_{xy}\left( f \right) }{\sqrt{S_{xx}\left( f \right) \cdot S_{yy}\left( f \right) }}\right) , \end{aligned}$$

where $S_{xx}$ and $S_{yy}$ are the individual power spectral density of *x* and *y*, and $S_{xy}$ is the cross-power spectral density of *x* and *y* at frequency *f*.

The imaginary part of the coherency has been used in conjunction with artificial neural networks (ANNs) to evaluate consciousness level of CLIS patients [42–44], and both methods have also been employed with the same goal with DoC patients [24, 45]. Periods of unresponsiveness in healthy subjects under anaesthesia are portrayed by a decrease of coherence in the $\delta$ bands, more particularly in the frontal and central electrodes [46]. Moreover, a global decrease of coherence is observed during ketamine-induced unconsciousness, while an increase of power and coherence in the higher frequencies is seen during recovery of consciousness [29].

Given the type of task presented to the patient and that $\theta$ band plays an important part in working memory [15], only the coherency in this frequency band will be used.

wSMI evaluates not only linear, but also non-linear relationships between two signals, channels or brain regions *x* and *y*. It quantifies information sharing between the two entities and is computed using Eq. (7) [24]. The signals are first transformed into series of discrete symbols $(\hat{x},\hat{y} )$, organised according to trends in amplitudes of *k* time samples separated by a temporal separation of elements $\tau$. wSMI equals 1 when the two signals are completely dependent, and equals 0 when they are entirely independent:

7 $$\begin{aligned} w{\text{SMI}}(x,y) = \frac{1}{\log (k!)}\sum _{\hat{x}\in \hat{X}}^{}\sum _{\hat{y}\in \hat{Y}}^{}w(\hat{x},\hat{y})p(\hat{x},\hat{y})\log \left( \frac{p(\hat{x},\hat{y})}{p(\hat{x})p(\hat{y})} \right) . \end{aligned}$$

Similar to the previous case, only wSMI in the $\theta$ band was used. Indeed, especially in that frequency band, wSMI was able to precisely assess the long-range connectivity patterns that are related to consciousness in theory [47]. In this case, $k=3$ and $\tau = 16$ ms to capture the patterns of the EEG in the $\theta$ frequencies [24]. Greater values represent higher levels of consciousness [48].

#### Consciousness level assessment

In this study, the degree to which a subject is conscious designates the consciousness level. Its value is determined by the outputs of the two soft clustering approaches: *Fuzzy c-means* (FCM) [49] and *Gaussian Mixture Models* (GMM) [50]. On one hand, FCM is the most used soft-clustering approach; and on the other hand, Gaussian model is the most used model in a model-based clustering [50]. Hard clustering partitions the data points into several disjointed clusters. Thus, each data point belongs to only one cluster. Soft-clustering analysis allows them to belong to multiple clusters with a certain degree of membership. The sum of the degrees of membership to all clusters equals 1.

##### FCM

FCM can be thought as the soft version of the *K*-means algorithm by introducing a fuzzy overlap $m>1$ [49]. Initially, the cluster memberships $\mu _{ij}$ are randomly attributed. Then the cluster centres $c_{ij}$ are calculated using Eq. (8):

8 $$\begin{aligned} c_{ij} = \frac{\sum _{i=1}^{D} \mu _{ij}^m x_i}{\sum _{i=1}^{D} \mu _{ij}^m}, \end{aligned}$$

where *m* is the fuzziness parameter ($m=1$ corresponds to a hard-clustering), $x_i$ is the *i*th data point. Afterwards, the cluster membership values $\mu _{ij}$ are updated using Eq. (9), and then the objective function $J_m$ is computed using Eq. (10). These steps are repeated until the objective function converges to a minimum, or when a maximum number of iterations are achieved:

9 $$\begin{aligned}{} & {} \mu _{ij} = \frac{1}{\sum _{k=1}^{N}\left( \frac{||x_i-c_j||}{||x_i-c_k||}\right) ^{\frac{2}{m-1}}}, \end{aligned}$$

10 $$\begin{aligned}{} & {} J_m = \sum _{i=1}^{D} \sum _{j=1}^{N} \mu _{ij}^m \Vert x_i - c_j \Vert ^2. \end{aligned}$$

The MATLAB function *|fcm| was used to perform the analysis with the following parameters: $m = 2$ [51], the maximum number of iterations is 1000, and the minimum improvement in the objective function between two consecutive iterations is $\epsilon = 1e^{-5}$.

##### GMM

GMM, on the other hand, is a model-based cluster analysis approach that uses a Gaussian mixture distribution $f\left( \textrm{x}_i / z_{ig}=1,\theta _g \right) \sim \mathcal {N}\left( \mu _g,\Sigma _g \right)$ as a model (Eq. 11) [52]. It is assumed that the data are produced by a random statistical model that the clustering method attempts to recover [50]. Given $\mathrm {x = (x_1,x_2,...,x_n)} \in \mathbb {R}^p$, the random vector $\textrm{x}_i$ is assumed to arise from a finite mixture of probability density functions:

11 $$\begin{aligned} f\left( \textrm{x}_i,\Theta \right) = \sum _{g=1}^{K}\pi _g\Phi \left( \textrm{x}_i / \mu _g,\Sigma _g \right) , \end{aligned}$$

where:

- *K* is the number of clusters. Each mixture component density is associated to a specific parametric class and represents a cluster.
- $\pi _g>0, (g = 1,...,K)$ and $\sum_{g=1}^{K}\pi _g=1$ are the mixing proportions.
- $\Phi =(\pi _1,...\pi _{g-1},\mu _1,...\mu _g, \Sigma _1,...,\Sigma _g)$ is the parameter vector.
- $\Phi \left( \textrm{x}_i / \mu _g,\Sigma _g \right)$ is the underlying component-specific density function with parameters $\mu _g,\sigma _g, g=1,...,K$. The parameters in $\Phi$ are estimated by the maximum likelihood optimisation, more precisely by using the iterative *Expectation-Maximisation* (EM) algorithm [50].

The model in Eq. (11) generates ellipsoidal clusters centred at the mean vector $\mu _g$, and $\sigma _g$ controls the other geometrical properties of each cluster. Difference of means in the different component models suggest that the model distinguishes among the *K* classes [53].

The EM algorithm consists of an E-step, during which it calculates posterior probabilities (conditional probability that is assigned after the relevant evidence is taken into account) of cluster memberships; and an M-step, during which it estimates the cluster parameters by applying maximum likelihood and using the cluster-membership posterior probabilities as weights. These steps are iterated until the algorithm converges to a local optimum. Once it reaches it, the soft partition is obtained by assigning each data point to the cluster with the highest posterior probability.

The MATLAB functions |fitgmdist| and |posterior| were used to perform the analysis with the same parameters as with FCM.

##### Ensemble average

The previously computed features were normalised and used as input vector to the clustering analysis. Its dimensionality is $N_{\text{samples}}~\times ~N_{\text{features}}$. Given that the aim is to distinguish between *conscious* and *unconscious* states, the number of clusters is then $N=2$. So accordingly, the consciousness level is indicated by the degree of memberships to the cluster representing *conscious* states. This means that an unconscious state is represented by a 0, while a value of 1 would imply conscious states. To obtain a final unique value that will determine the patients’ consciousness levels, the results of FCM and GMM were averaged using Eq. (12):

12 $$\begin{aligned} P_{\text{avg}}(c,m_1 m_2) = {\text{avg}}(P(c,m_1), P(c,m_2)), \end{aligned}$$

where $P(c,m_1)$ (resp. $P(c,m_2)$) is the probability that the object *i* is a member of cluster *c* in partition $m_1$ ($m_2$ resp.).

## Results

Results can be grouped into two categories: a group for which the proposed method was functional, and another one for which said approach was amiss. An example from each category will be showcased in this section, respectively, those of patients L1 and S7. The results for the remaining patients are presented in Additional file 1.

### Patient L1

#### Estimation of the patients’ consciousness levels

**Fig. 4:**
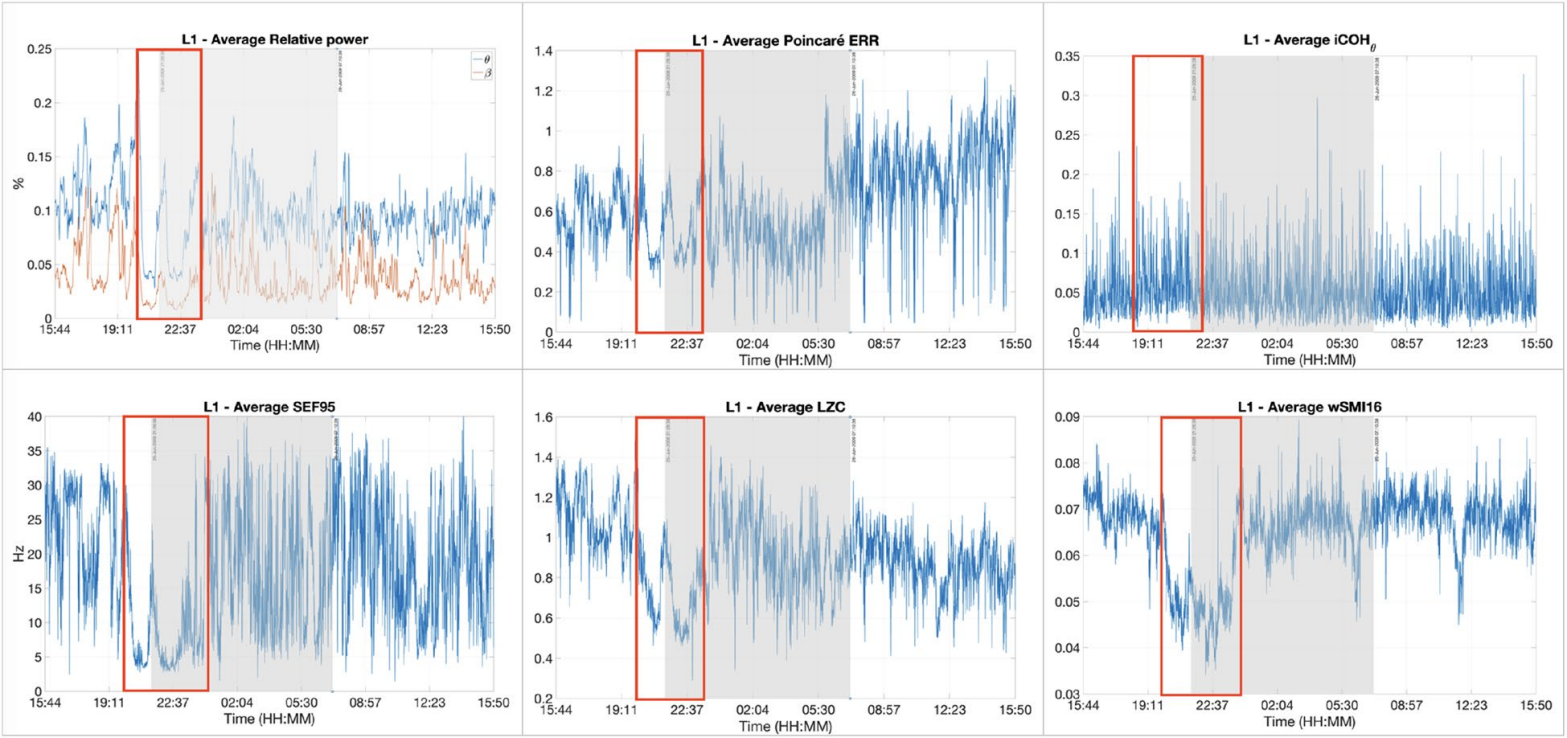
Different features for patient L1. From left to right and then from top to bottom: relative powers of $\theta$ and $\beta$, ERR, iCOH, SEF95, LZC and wSMI resp. represented in the *y*-axis. The recording lasted for around 24 h: from 15:44 until 15:50 the next day. The shaded areas represent the night time. Higher values of each feature are representative of higher levels of consciousness and inversely. The different features showed similar results, particularly a noticeable drop between the time frame delimited by the red rectangles in the figure, indicating a definite decrease of consciousness. The patient was certainly unconscious then

Patient L1 is a 21-year-old patient in a VS following a traumatic brain injury (TBI) that happened 7 months before the data recording. This patient was the only one which EEG features produced practically concurring results, although not clearly visible for iCOH. These results can be seen in Fig. 4. Additionally, patient L1 also possesses the most eyes scoring information.

The results of this particular patient illustrate the perfect case in which all results are mostly consistent with one another. In other words, their values increase or decrease at the same time. A higher consciousness state is characterised by a larger value of each specific feature, and inversely. For example, the definite decrease observed during the time frame delimited by the red rectangle in Fig. 4 indicates that the patient was certainly unconscious. Likewise, the higher values observed before the same time frame may indicate that the patient was conscious.

**Fig. 5:**
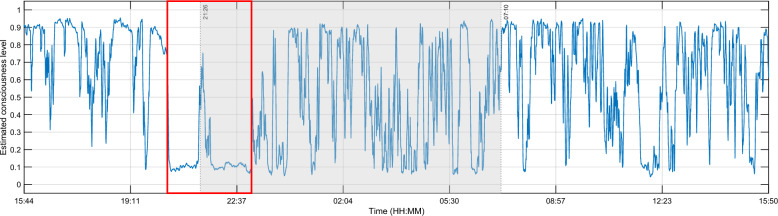
Estimated consciousness level for VS patient L1 obtained with the average ensemble. The *x*-axis represents the time, and the y-axis the level of consciousness. 0 corresponds to *unconscious* and 1 represents *conscious*. The shaded area represents night time. The decrease observed inside the red rectangle with the different features were effectively observed here in the estimated consciousness level

The two clustering analysis (FCM and GMM) previously introduced were then applied to the input vector consisting of all the calculated features. They obtained results were subsequently averaged using Eq. (12). Figure 5 illustrates this averaging for patient L1. This result is consistent with the observations on each unique feature. As already mentioned earlier, the degree of membership to the *conscious* cluster is chosen as the consciousness level of the patients. Accordingly, the patient was undoubtedly conscious during most the recordings, but was unconscious in particular during the same time frame delimited by the red rectangle in Fig. 4.

**Fig. 6:**
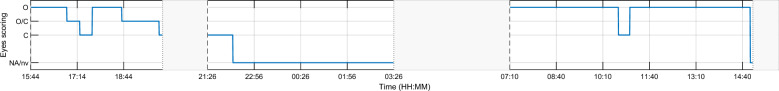
Eyes scoring of patient L1. The time is represented in the *x*-axis. On the *y*-axis: O: eyes open, C: eyes closed. O/C: intermittent opening and closing of the eyes, *Na*/*nv*: scoring unavailable due to some technical problems. The blank areas are the time frames during which no eyes scoring were recorded

Figure 6 presents the eyes scoring of patient L1. A comparison between these scores and the estimated consciousness levels shows that open eyes were observed during the times when the algorithm estimated higher consciousness levels, and most closed eyes were detected during the times with lower consciousness levels.

#### Features contributions

Table 2 shows the contribution of each feature to the final estimation for patient L1. The values were determined with a Spearman correlation [54]. SEF95 is the measure that contributes the most with a correlation of 0.9039, followed by $P_{\text{beta}}$ with 0.8571. On the other hand, for this patient, the connectivity features iCOH and wSMI were the less contributing ones.

**Table 2:** Spearman correlation coefficients for VS patient L1 between all features and estimated levels of consciousness

	FCM	GMM	Ensemble
$P_{theta}$	0.5016	0.6331	0.5159
$P_{beta}$	0.8407	0.9449	0.8571
SEF95	0.8928	0.9579	0.9039
ERR	0.5391	0.5433	0.5350
LZC	0.7254	0.8003	0.7385
iCOH	0.0062	0.0197	0.0058
wSMI	0.2963	0.2782	0.2888

The cells in grey represent the correlation coefficients with $p > 0.05$

**Fig. 7:**
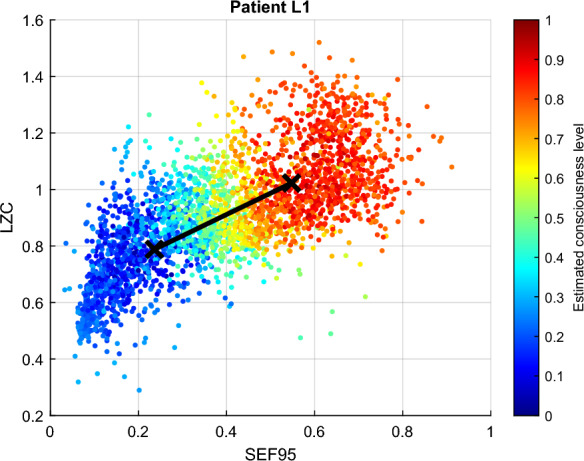
FCM clustering results for patient L1: largest inter-cluster difference. In an ideal case, data points representing unconscious states are located in the lower left part of the plot, while those indicating conscious states are located in the top right part

**Fig. 8:**
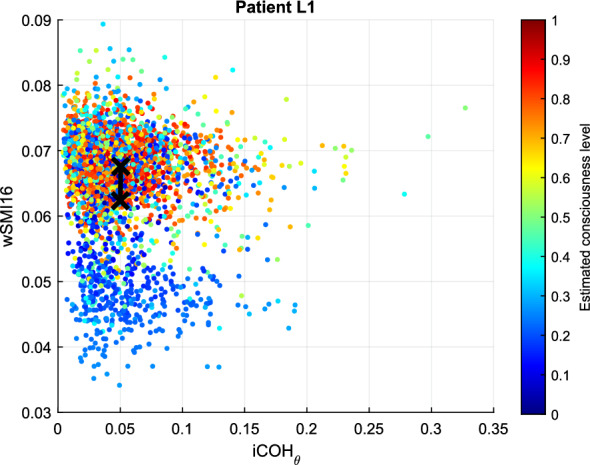
FCM clustering results for patient L1: smallest inter-cluster difference. In an ideal case, data points representing unconscious states are located in the lower left part of the plot, while those indicating conscious states are located in the top right part

The FCM clustering results between the two most and the two least contributing features are illustrated in Figs. 7 and 8. Unconsciousness is represented in blue, while consciousness is in red. Ideally, the data points representing unconscious states are located in the left bottom area of the plot, while the conscious states are situated on the top right area of the plot. In Fig. 7, which shows the two EEG characteristics with the largest inter-cluster differences, the degree of membership of the data points progresses smoothly from blue (bottom left of the plot) to red (top right of the plot), i.e. from unconscious to conscious states.

Figure 8 illustrates the two features with the worst inter-cluster distance. It can be seen that in this case, data points with different degree of membership values to the *conscious* cluster are intermingled. Most data points representing low degrees of membership are still located in the lower part of the plots, as it should be. However, the upper part contains objects with different degrees of membership. It can be inferred from these result that the best features for patient L1 are LZC and SEF95, while the connectivity measures produced non-distinguishable clusters for both clustering analysis methods.

### Patient S7

Patient S7 is a 30-year-old patient in an MCS. His condition also results from a TBI that occurred 120 months before the data recording. This is the longest time since injury across in this group of patients. Apart from that, patient S7 also exhibited the lowest centroid linkage distance (separation between two objects belonging to two different clusters, computed with the Euclidean distance) in the clustering results.

#### Consciousness levels estimations

**Fig. 9:**
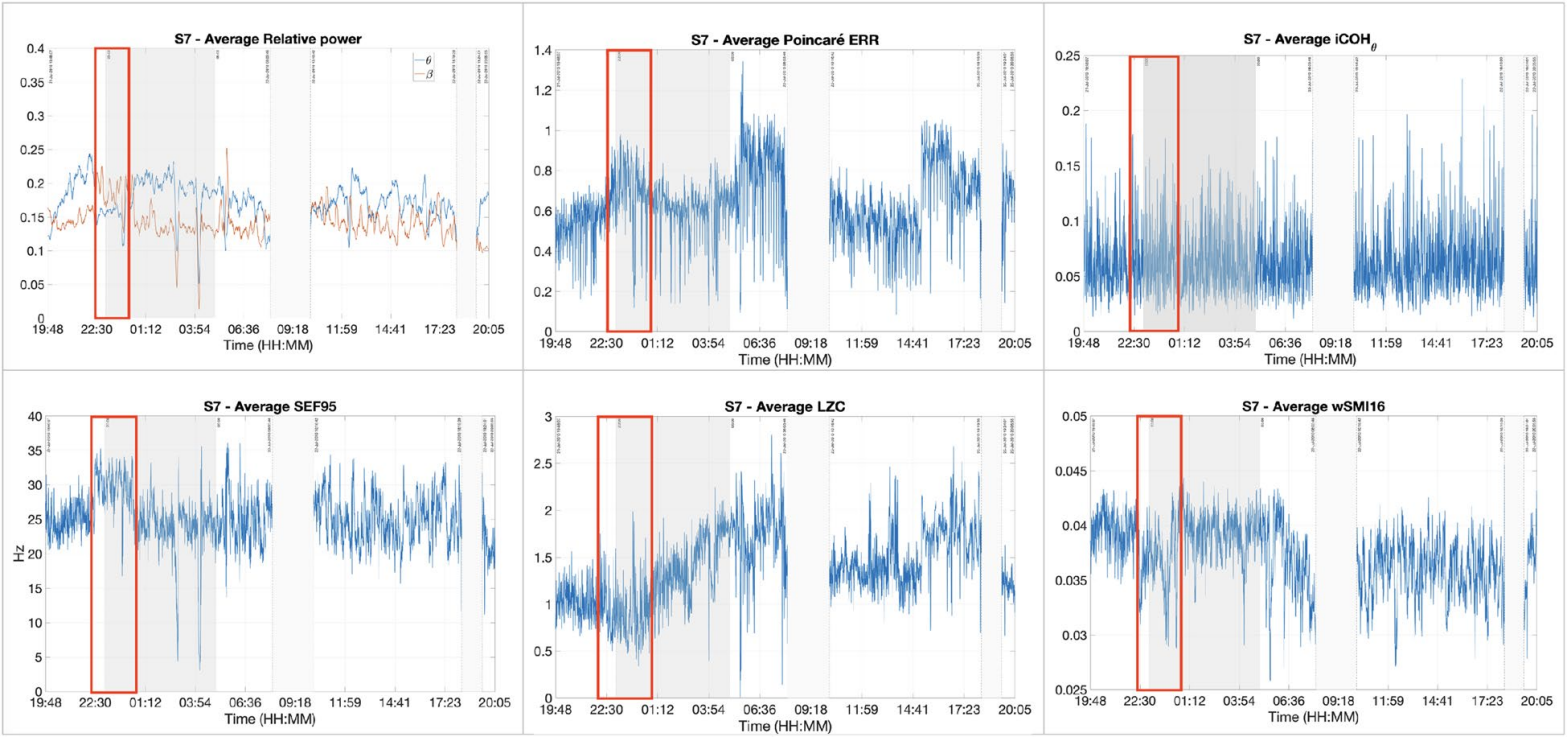
Different features for patient S7. From left to right and then from top to bottom: relative powers of $\theta$ and $\beta$, ERR, iCOH, SEF95, LZC and wSMI. The recording lasted for around 24 h: from 15:44 until 15:50 the next day. The shaded areas represent the night time. The different features showed diverging results. For example, a drop/increase between the time frame delimited in red. Higher values of each feature are representative of higher levels of consciousness and inversely. Given the values of the individual features throughout the whole recording, it can be inferred that the patient was certainly conscious all along

Figure 9 illustrates the results obtained from the different features for patient S7. It shows that the results of the individual EEG signatures are sometimes divergent. For example, results obtained from the spectral features and the complexity measures are similar, but are differing from those of the connectivity measures. When an increase is observed on the former group, a decrease is detected on the latter, and inversely. For example, while an increase of the $\theta$ and $\beta$ relative powers, SEF95, and ERR was observed between 22:25 and 00:40, LZC and wSMI values were dropping. Normally, low values suggest a reduced consciousness level. However, considering the values of the features, especially the SEF (above the alpha band, represented in green in Fig. 9) and the complexity measures ($\ge 1$) in particular, it can be inferred that the patient was conscious during most of the recording.

**Fig. 10:**
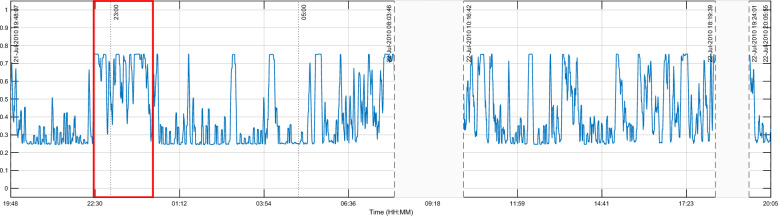
Estimated consciousness level for patient S7 obtained with the average ensemble. The gray area represents night time. The *x*-axis represents the time. In the *y*-axis, 0: unconscious, 1: conscious

**Fig. 11:**
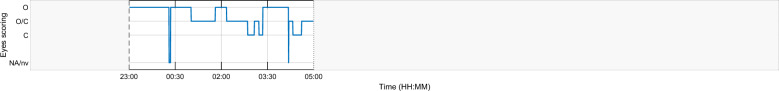
Eyes scoring of patient S7. The *x*-axis represents the time. On the *y*-axis: O: eyes open, C: eyes closed. O/C: intermittent opening and closing of the eyes, *Na*/*nv*: scoring unavailable due to some technical problems. The blank areas represent the time frames during which no eyes scoring were recorded. Eyes scoring was available only during night time (from 23:00 to 05:00)

Figure 10 illustrates the results of the ensemble clustering analysis. An increase can be seen during the same time frame delimited in the red rectangles in Fig. 9, when compared to the estimated consciousness levels. It appears as the algorithm performed a kind of majority upon with the results of the different features. Figure 11 shows the eyes scoring of MCS patient S7. The scoring was available only from 23:00 to 05:00.

#### Features contributions

Table 3 displays the contribution of each feature to the final level of consciousness estimation for patient S7. First of all, $P_{\text{theta}}$ and wSMI contributed highly but negatively to the final result. The other features, on the other hand, are positively but only moderately correlated with the estimated consciousness levels. Furthermore, LZC and iCOH for FCM, and ERR and LZC, which are, respectively, the pairs of features with the lowest inter-cluster distances, are also the EEG characteristics that correlated the less to the estimated consciousness levels.

**Table 3:** Spearman correlation coefficients for MCS patient S7 between all features and estimated levels of consciousness

	FCM	GMM	Ensemble
$P_{\text{theta}}$	− 0.5660	− 0.7500	− 0.7181
$P_{\text{beta}}$	0.3920	0.3223	0.3550
SEF95	0.5771	0.4907	0.5294
ERR	0.3611	0.1181	0.1655
LZC	0.3113	0.0547	0.0910
iCOH	0.1535	0.1283	0.1330
wSMI	− 0.7443	− 0.7511	− 0.7423

The cells in grey represent the correlation coefficients with $p > 0.05$

**Fig. 12:**
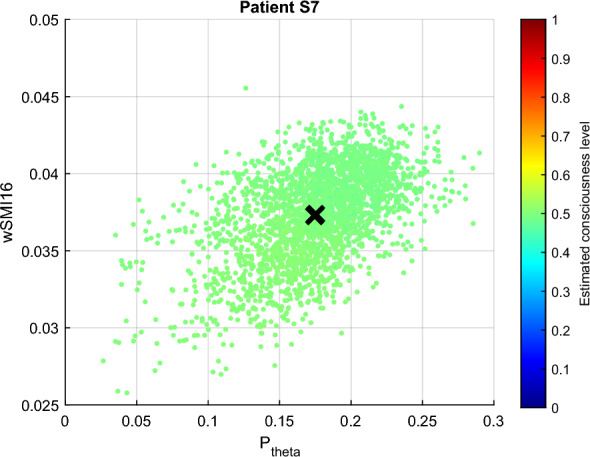
FCM clustering results representing the largest inter-cluster differences for patient S7. Unconsciousness is represented in blue, while consciousness is in red

**Fig. 13:**
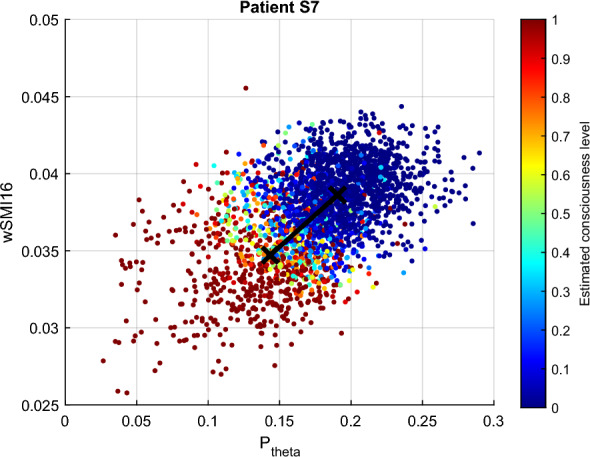
GMM clustering results representing the largest inter-cluster differences for patient S7. Unconsciousness is represented in blue, while consciousness is in red

The clustering results between the two most contributing features obtained from FCM and GMM are illustrated in Figs. 12 and 13. In Fig. 12, the *conscious* and *unconscious* clusters obtained from FCM are practically indistinguishable, with a very low inter-cluster difference of 0.0067. The dissimilarities were computed on the normalised features. Additionally, the average degree of membership to the *conscious* cluster of all the data points is 0.4979 (green colour in the figure). The clustering analysis results also showed that $i{\text{COH}}_{\theta }$ and LZC display the lowest inter-cluster difference with 0.0019. Both values are extremely low, so in the figure, they are practically overlapping.

The degrees of membership obtained from the GMM clustering analysis cover more value ranges, although there was no smooth transition from unconscious to conscious states. As seen in Fig. 9 and Table 3, $P_{\text{theta}}$ and wSMI contradict those of the other features. Particularly, low values of wSMI and $P_{\text{theta}}$ belong to the *conscious* cluster, and inversely. These observations are also contradicting the hypothesis established in the previous section. wSMI and $P_{\text{theta}}$ also display the largest inter-cluster distance with a value of 0.2662. The lowest distance is observed between LZC and ERR with 0.0125.

### Performance of the approach

Overall, the proposed approach was able to estimate consciousness levels of 20 of the 23 patients. In general, analogous to a majority vote, the estimations of consciousness levels from both FCM and GMM are positively correlated with the majority of the individual features. In other words, the approach was able to convey the increases and decreases of the patients’ levels of consciousness from them. On the other hand, the accuracy of these estimations depends on the overall inter-cluster differences. First of all, the levels of consciousness values were highly influenced by the features with the largest inter-cluster distance and vice versa. The correlation coefficients between the features and the estimated levels of consciousness from the clustering analysis are reported in Additional file 1.

The results showed that there is no common best or worst feature shared by all patients. Each individual is different, and so are the most and less efficient features for each of them. In addition, when the dissimilarities are large enough, the estimated levels of consciousness are remarkably accurate when matched with the outcomes of each individual measure (as is the case of patient L1). However, when it is not the case, i.e. the inter-cluster distances are small, the estimations are not correctly conveyed. This latter case was observed for patients L13 and S13 in addition to patient S7 which case was presented in the following section.

To further evaluate the performance of the proposed approach, the obtained estimations of consciousness levels were binarised and compared to the eyes states. The values below the threshold were set to 0 and those above it, to 1. Similarly, values of 1 were assigned to open eyes (“O”), and 0 was appointed to periods of closed eyes (“C”). Accordingly, only the data with available eyes scoring were used. The threshold values range from 0.3 to 0.7 with a 0.1 increment, given that 0.5 could not possibly mean the separation between conscious and unconscious states. The performance accuracy was computed for all threshold values using Eq. (13), in which *TP*: true positive, *TN*: true negative, *FP*: false positive, and *FN*: false negative:

13 $$\begin{aligned} {\text{Accuracy}} = \frac{{\text{TP+TN}}}{{\text{TP+TN+FP+FN}}}. \end{aligned}$$

**Fig. 14:**
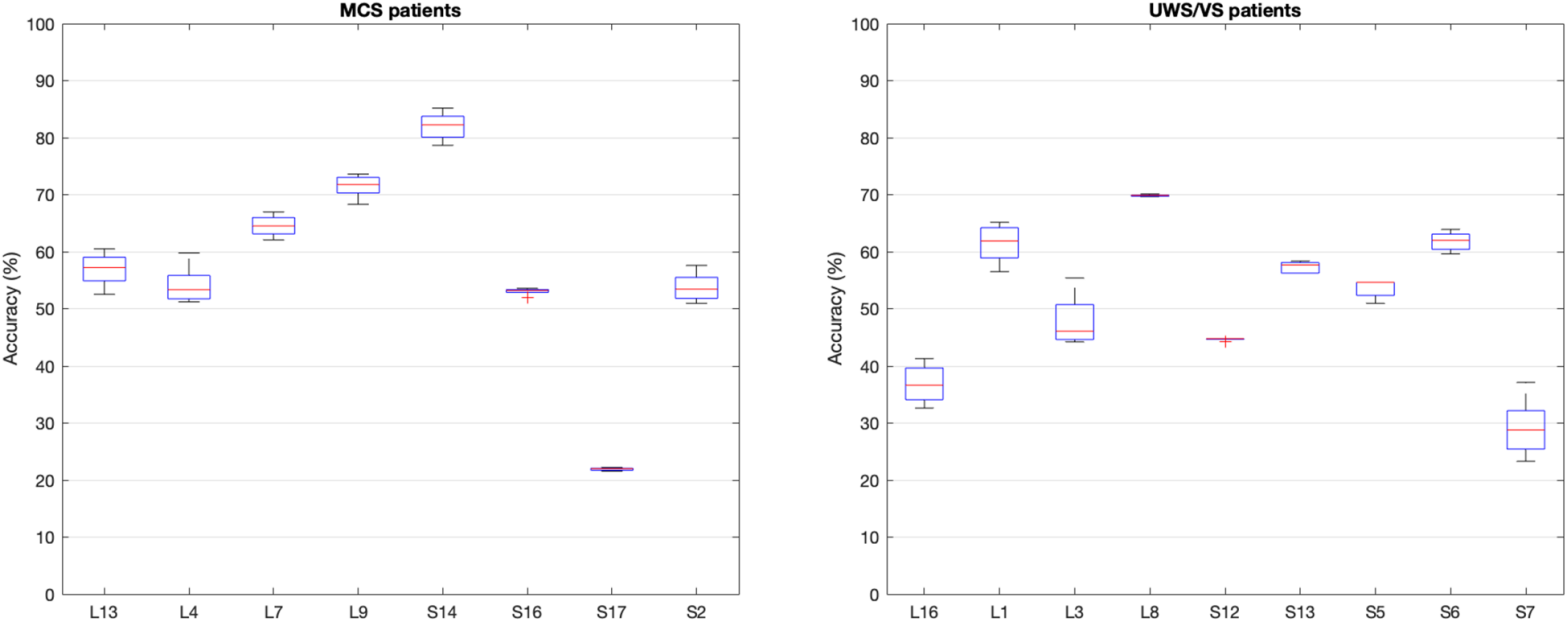
Performance of the approach. Overall, highest accuracies were obtained by MCS patients

Figure 14 shows the obtained results. On average, MCS patients achieved the highest accuracies for the different threshold values. However, one of the MCS patients (S17) also achieved the lowest accuracy with 22.22%. The highest accuracy for VS patients was achieved by patient L8 with 70.11%.

## Discussion

In this paper, a method to assess patients consciousness levels using a soft clustering analysis of a feature vector consisting of the combination of several EEG signatures is presented. The idea behind integrating multiple features was to increase the chances of detecting hidden characteristics that were missed by the other features, consequently maximising the probability of correctly estimating the patients’ actual state. The different features used in this work were weighted equally. Each of the features extracts a particular characteristic of the EEG signal. This work was based on the hypothesis that conscious states are defined by an increase of each of these signal attributes. Considering that the individual features may give conflicting results, the clustering analyses appear to find a consensus that conveys their combined variations. The results obtained from all patients suggest that the approach proposed in this paper works best when the data cover all possible consciousness states. This constitutes a major challenge considering the difficulty to record such patients’ data.

The obtained results are also consistent with the eyes scoring when available, i.e. conscious states were predicted mostly during periods of opened eyes, and inversely. First, eyes closed do not necessarily mean unconscious and inversely. However, it can be assumed that eyes closed correspond to unconsciousness since most of the scoring were done at night. Indeed, studies about DoC patients’ sleep patterns showed that open and closed eyes indicate periods of circadian sleep–wake [9, 10, 55]. More precisely, for this particular dataset, MCS patients showed increased values of high-to-low frequency power ratio and permutation entropy during the day, although no changes were detected for the VS patients [10]. Moreover, MCS patients also display sleep behaviour comparable to that of healthy subjects using permutation entropy on their EEG signals. This was not observed for VS patients [9]. Consequently, at least for the MCS patients, this shows the efficiency of the approach.

## Conclusion

An approach to evaluate patients’ consciousness levels was described in this paper. The ultimate aim is to apply it to EEG data recorded from CLIS patients. Nevertheless, in this study it was tested on data from DoC patients under the assumption that if it works for them, it will presumably also work for CLIS patients. This assumption was made considering that locked-in state is not a disorder of consciousness, and that according to previous research, CLIS patients preserve their cognitive functions. This has been proven in a study including one CLIS patients [56]. The results show that the presented method was able to depict the different increases and decreases of the chosen EEG signatures, accurately determining the consciousness levels of most of the patients. The accuracy of the estimated level depends on the distance between the clusters centroids, i.e. there should be enough data so that all possible states (from *unconscious* to *conscious*) are represented.

All features were weighted equally during the analysis and no feature selection was performed. Given that some features may be more relevant than others for each individual, future work will primarily focus on tailoring them to each patient. Moreover, additional EEG characteristics can be included to gather more hidden patterns and improve the system. Likewise, other soft-clustering approaches could also be investigated in place of FCM and GMM. The approach proposed here can be used as an additional tool to the traditional behavioural tests to help clinicians reduce the misdiagnosis rate, especially for (completely) locked-in patients.

## Supplementary Information

**Additional file 1.** Consciousness levels results for all DoC patients.

## Data Availability

Restrictions apply to the availability of these data. Data were obtained from Dr. Manuel Schabus from the University of Salzburg, Austria.

## Abbreviations

BCI: Brain–computer interface
CLIS: Completely locked-in syndrome
CRS-R: Coma Recovery Scale-Revised
CVA: Cerebrovascular accident
DoC: Disorders of consciousness
ECoG: Electrocorticogram
EEG: Electroencephalogram
EM: Expectation-maximisation
ERR: Ellipsoid radius ratio
FCM: Fuzzy c-means
GMM: Gaussian mixture models
iCOH: Imaginary part of coherency
LZC: Lempel–Ziv complexity
MCS: Minimally Conscious State
PSD: Power spectral density
REM: Rapid eye movement
RP: Relative power
SEF: Spectral edge frequency
SSPE: Subacute sclerosing panencephalitis
TBI: Traumatic brain injury
UWS: Unresponsive wakefulness syndrome
VS: Vegetative state
wSMI: Weighted symbolic mutual information
