# Supplementary material for: Assessing consciousness in patients with disorders of consciousness using soft-clustering
Source: Brain Inform. 2023 Jul 14;10(1):16. doi: 10.1186/s40708-023-00197-5 (PMC10348975; doi:10.1186/s40708-023-00197-5)
Supplement: Supplementary file 1 — Additional file 1. Consciousness levels results for all DoC patients. [file 40708_2023_197_MOESM1_ESM.pdf]

**Assessing Consciousness in Patients with Disorders of Consciousness using Soft-Clustering** – Supplementary file

Sophie Adama, Martin Bogdan

adama@informatik.uni-leipzig.de

## VS patients

### *Patient L3*

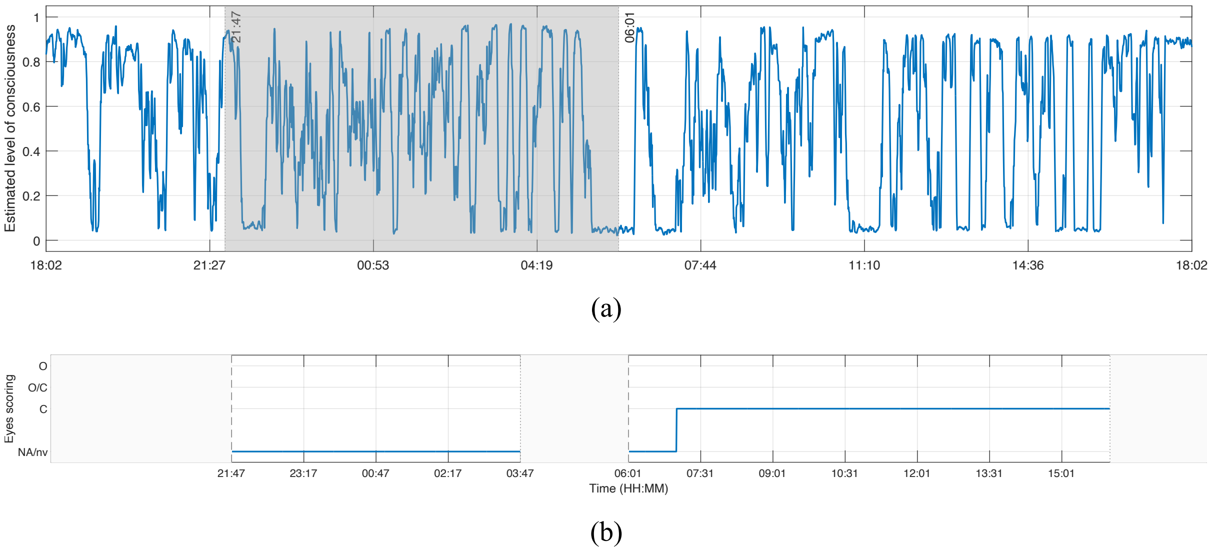


*Figure S 1: Estimated consciousness level (a) and Eyes scoring (b).*

*Table S 1: Spearman correlation coefficients between the features and the estimated levels of consciousness.*

| Features | FCM | GMM | Ensemble |
|---|---|---|---|
| $P_{theta}$ | 0,8615 | -0,8036 | 0,8627 |
| $P_{beta}$ | 0,8299 | -0,9717 | 0,8439 |
| SEF95 | 0,7900 | -0,9555 | 0,8059 |
| ERR | 0,7780 | -0,7568 | 0,7752 |
| LZC | 0,3736 | -0,4113 | 0,3803 |
| $iCOH_{theta}$ | 0,0144 | 0,0064 | 0,0082 |
| wSMI | -0,0978 | -0,0773 | -0,0866 |

## *Patient L13*

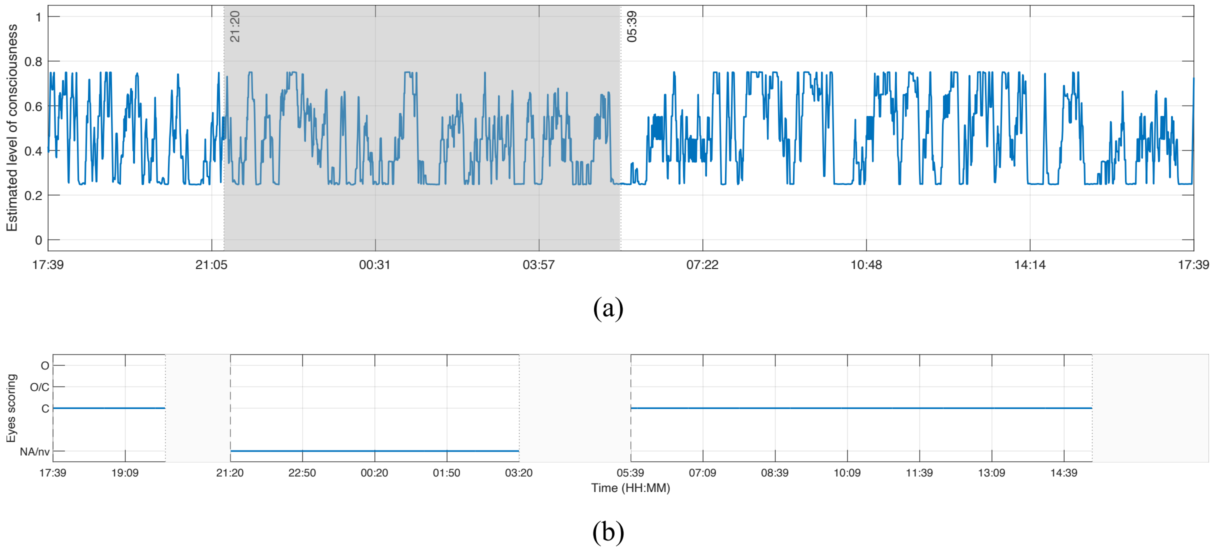


*Figure S 2: Estimated consciousness level (a) and Eyes scoring (b).*

*Table S 2: Spearman correlation coefficients between the features and the estimated levels of consciousness.*

| **Features** | **FCM** | **GMM** | **Ensemble** |
|---|---|---|---|
| $P_{theta}$ | 0,4933 | -0,5122 | 0,5354 |
| $P_{beta}$ | 0,4556 | -0,8997 | 0,8270 |
| SEF95 | 0,5011 | -0,90091 | 0,8481 |
| ERR | 0,0481 | -0,3973 | 0,3046 |
| LZC | -0,2004 | -0,2662 | 0,1242 |
| $iCOH_{theta}$ | 0,5246 | -0,0680 | 0,2032 |
| wSMI | 0,2718 | -0,3499 | 0,3227 |

## *Patient S12*

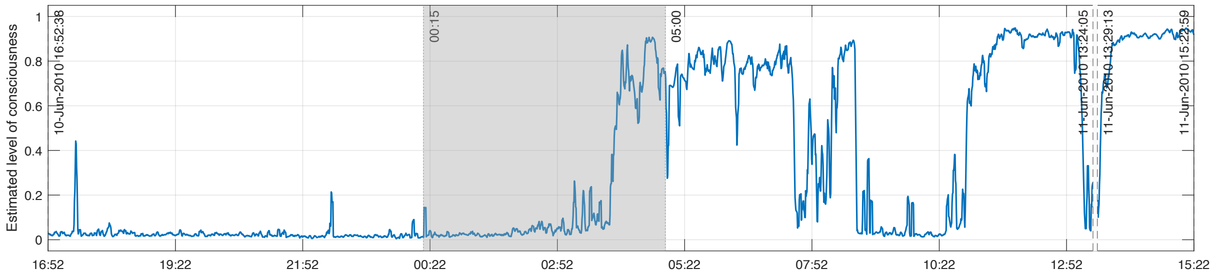


(a)

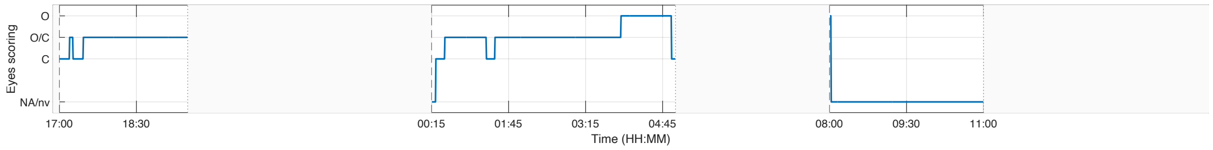


(b)

*Figure S 3: Estimated consciousness level (a) and Eyes scoring (b).*

*Table S 3: Spearman correlation coefficients between the features and the estimated levels of consciousness.*

| Features | FCM | GMM | Ensemble |
|---|---|---|---|
| $P_{theta}$ | -0,6193 | 0,5232 | 0,4908 |
| $P_{beta}$ | 0,4830 | 0,7516 | 0,6979 |
| SEF95 | 0,6922 | 0,8228 | 0,7358 |
| ERR | 0,7307 | 0,4314 | 0,5019 |
| LZC | 0,5034 | -0,0451 | -0,0058 |
| $iCOH_{theta}$ | -0,0026 | 0,3858 | 0,4479 |
| wSMI | 0,4503 | 0,2183 | 0,3042 |

## *Patient S13*

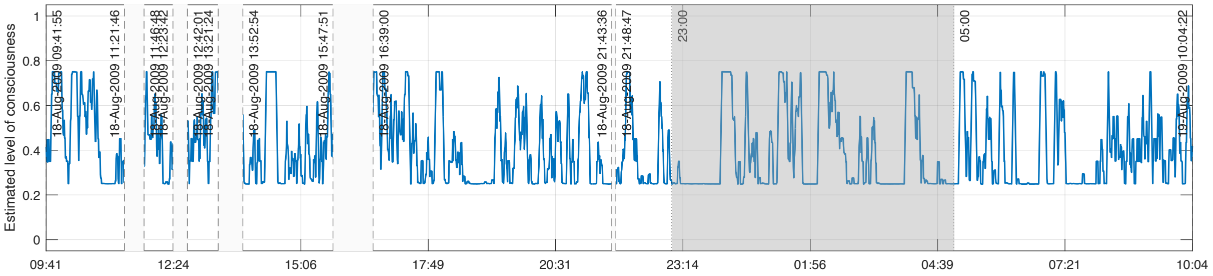


(a)

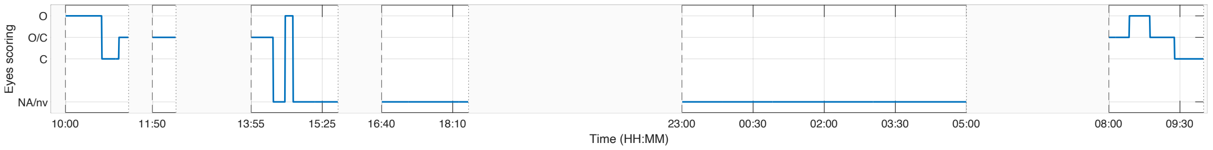


(b)

*Figure S 4: Estimated consciousness level (a) and Eyes scoring (b).*

*Table S 4: Spearman correlation coefficients between the features and the estimated levels of consciousness.*

| **Features** | **FCM** | **GMM** | **Ensemble** |
|---|---|---|---|
| $P_{theta}$ | 0,4820 | 0,2183 | 0,3042 |
| $P_{beta}$ | 0,5345 | 0,8942 | 0,8551 |
| SEF95 | 0,4645 | 0,9380 | 0,8732 |
| ERR | 0,3159 | 0,8638 | 0,7653 |
| LZC | -0,0988 | 0,7039 | 0,4809 |
| $iCOH_{theta}$ | 0,1504 | -0,0374 | 0,0237 |
| wSMI | 0,6432 | 0,0865 | 0,2793 |

## *Patient S14*

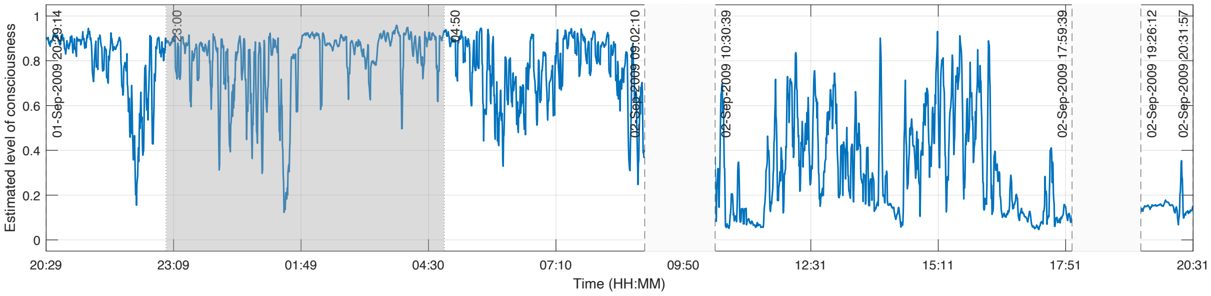


(a)

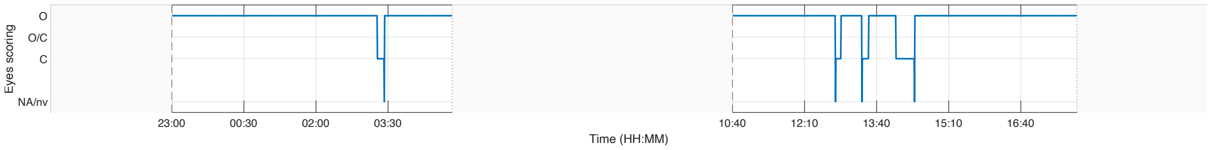


(b)

*Figure S 5: Estimated consciousness level (a) and Eyes scoring (b).*

*Table S 5: Spearman correlation coefficients between the features and the estimated levels of consciousness.*

| **Features** | **FCM** | **GMM** | **Ensemble** |
|---|---|---|---|
| $P_{theta}$ | -0,0744 | -0,0742 | -0,0307 |
| $P_{beta}$ | 0,8034 | -0,9272 | 0,8274 |
| SEF95 | 0,8454 | -0,9667 | 0,8713 |
| ERR | 0,7785 | -0,9244 | 0,8046 |
| LZC | 0,4942 | -0,3605 | 0,4553 |
| $iCOH_{theta}$ | 0,0416 | -0,0924 | 0,0483 |
| wSMI | 0,4954 | -0,5486 | 0,4988 |

### *Patient S16*

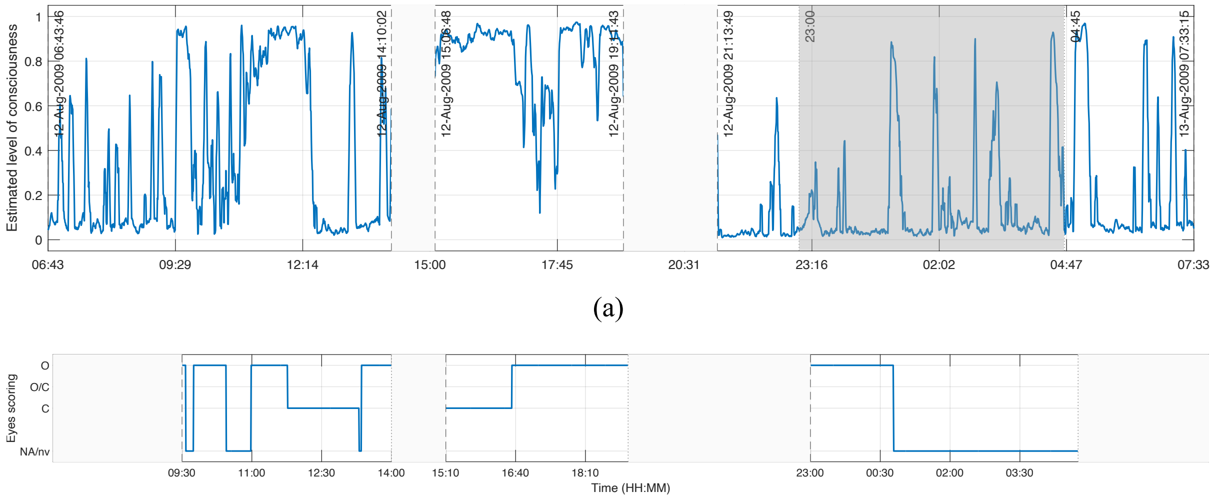


(a)

(b)

*Figure S 6: Estimated consciousness level (a) and Eyes scoring (b).*

*Table S 6: Spearman correlation coefficients between the features and the estimated levels of consciousness.*

| Features | FCM | GMM | Ensemble |
|---|---|---|---|
| $P_{theta}$ | -0,4848 | -0,7251 | -0,4893 |
| $P_{beta}$ | 0,8016 | 0,8008 | 0,8273 |
| SEF95 | 0,8004 | 0,7701 | 0,8269 |
| ERR | 0,3968 | 0,3170 | 0,3861 |
| LZC | 0,6407 | 0,7669 | 0,6729 |
| $iCOH_{theta}$ | 0,0738 | 0,0423 | 0,0719 |
| wSMI | -0,4714 | -0,7052 | -0,4821 |

## *Patient S17*

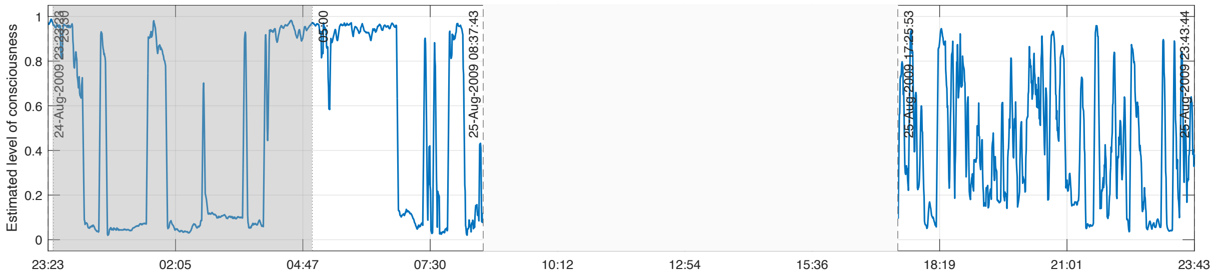


(a)

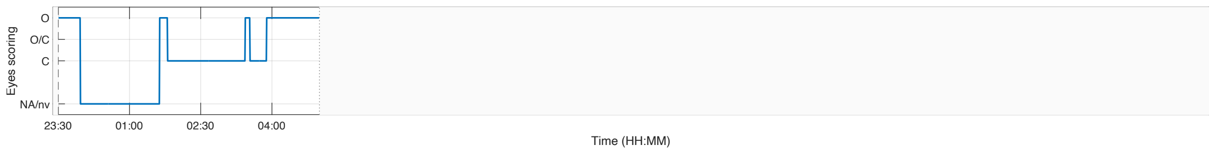


(b)

*Figure S 7: Estimated consciousness level (a) and Eyes scoring (b).*

*Table S 7: Spearman correlation coefficients between the features and the estimated levels of consciousness.*

| Features | FCM | GMM | Ensemble |
|---|---|---|---|
| $P_{theta}$ | -0,6990 | 0,7421 | -0,6779 |
| $P_{beta}$ | 0,5480 | -0,6529 | 0,5850 |
| SEF95 | 0,8449 | -0,8931 | 0,8583 |
| ERR | 0,5551 | -0,6222 | 0,5681 |
| LZC | 0,7808 | -0,7316 | 0,7612 |
| $iCOH_{theta}$ | -0,0219 | 0,0338 | -0,0205 |
| wSMI | -0,7970 | 0,7950 | -0,7971 |

## MCS patients

### *Patient L4*

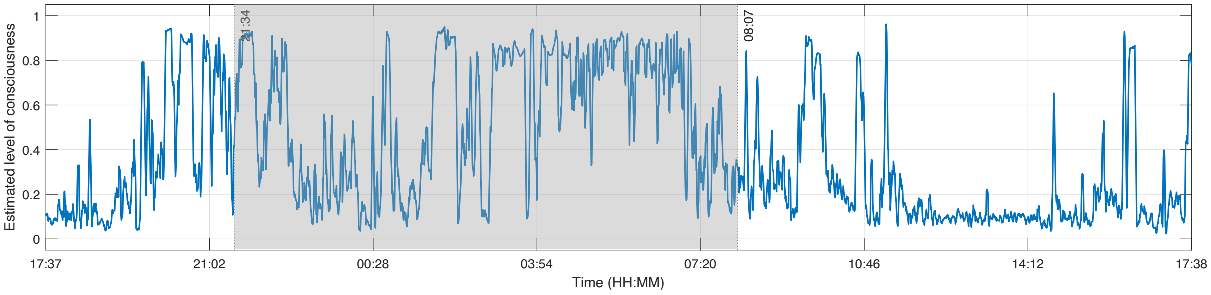


(a)

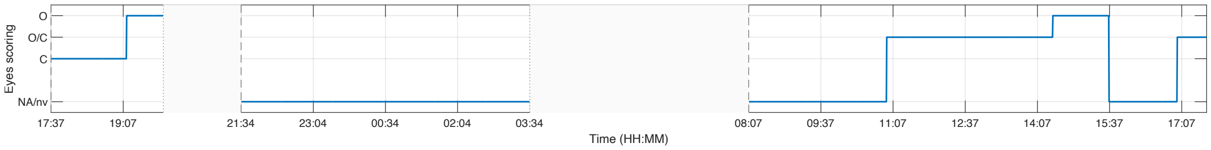


(b)

*Figure S 8: Estimated consciousness level (a) and Eyes scoring (b).*

*Table S 8: Spearman correlation coefficients between the features and the estimated levels of consciousness.*

| **Features** | **FCM** | **GMM** | **Ensemble** |
|---|---|---|---|
| $P_{theta}$ | 0,6894 | 0,5700 | 0,6204 |
| $P_{beta}$ | 0,8503 | 0,9447 | 0,8673 |
| SEF95 | 0,8212 | 0,9506 | 0,8519 |
| ERR | 0,4857 | 0,4242 | 0,4528 |
| LZC | 0,7665 | 0,9396 | 0,8038 |
| $iCOH_{theta}$ | -0,0261 | -0,0589 | -0,0282 |
| wSMI | 0,1958 | 0,3729 | 0,2592 |

### *Patient L7*

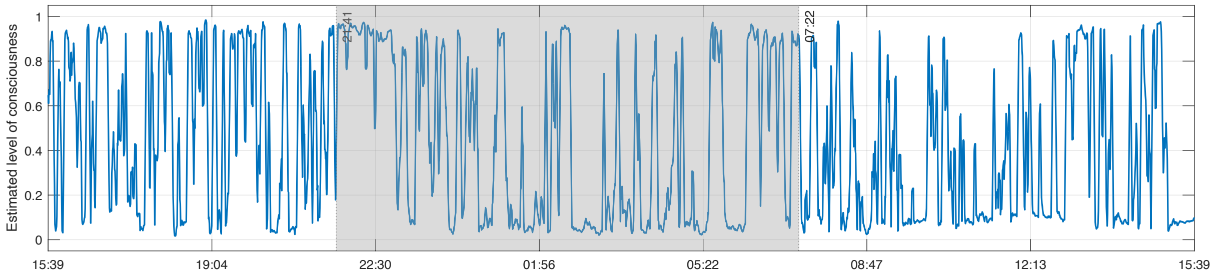


(a)

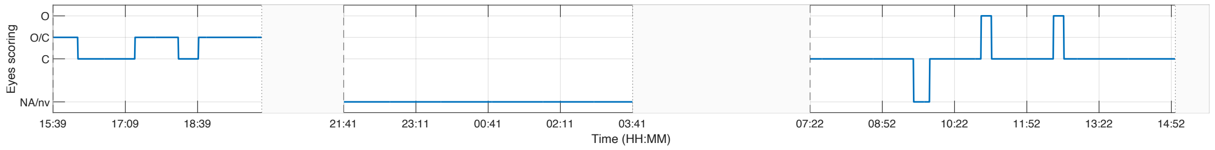


(b)

*Figure S 9: Estimated consciousness level (a) and Eyes scoring (b).*

*Table S 9: Spearman correlation coefficients between the features and the estimated levels of consciousness.*

| Features | FCM | GMM | Ensemble |
|---|---|---|---|
| $P_{theta}$ | -0,2871 | 0,2455 | -0,2858 |
| $P_{beta}$ | 0,8024 | -0,9249 | 0,8042 |
| SEF95 | 0,8626 | -0,9847 | 0,8638 |
| ERR | 0,0587 | 0,0849 | 0,0585 |
| LZC | 0,7572 | -0,9060 | 0,7583 |
| $iCOH_{theta}$ | -0,0283 | 0,0630 | -0,0282 |
| wSMI | 0,7688 | -0,8472 | 0,7685 |

## *Patient L8*

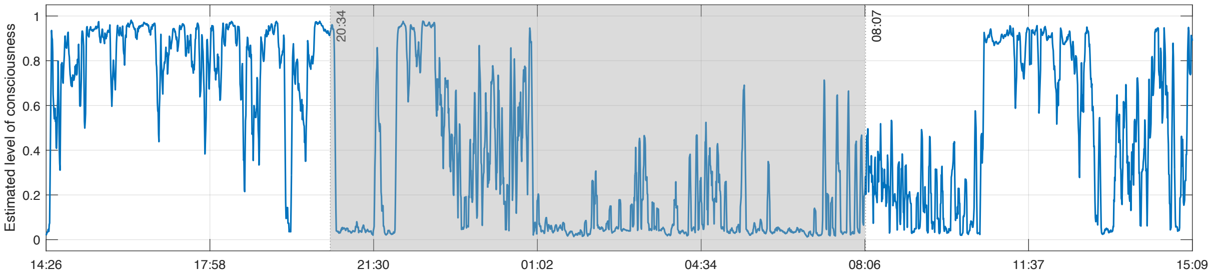


(a)

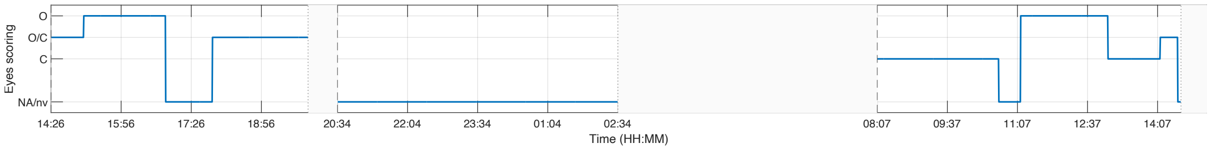


(b)

*Figure S 10: Estimated consciousness level (a) and Eyes scoring (b).*

*Table S 10: Spearman correlation coefficients between the features and the estimated levels of consciousness.*

| Features | FCM | GMM | Ensemble |
|---|---|---|---|
| $P_{theta}$ | -0,1053 | 0,2060 | -0,1166 |
| $P_{beta}$ | 0,8685 | -0,9443 | 0,8758 |
| SEF95 | 0,8782 | -0,9440 | 0,8866 |
| ERR | 0,8664 | -0,9556 | 0,8773 |
| LZC | 0,8440 | -0,9398 | 0,8553 |
| $iCOH_{theta}$ | 0,0566 | -0,0348 | 0,0504 |
| wSMI | 0,1601 | -0,2636 | 0,1621 |

### *Patient L9*

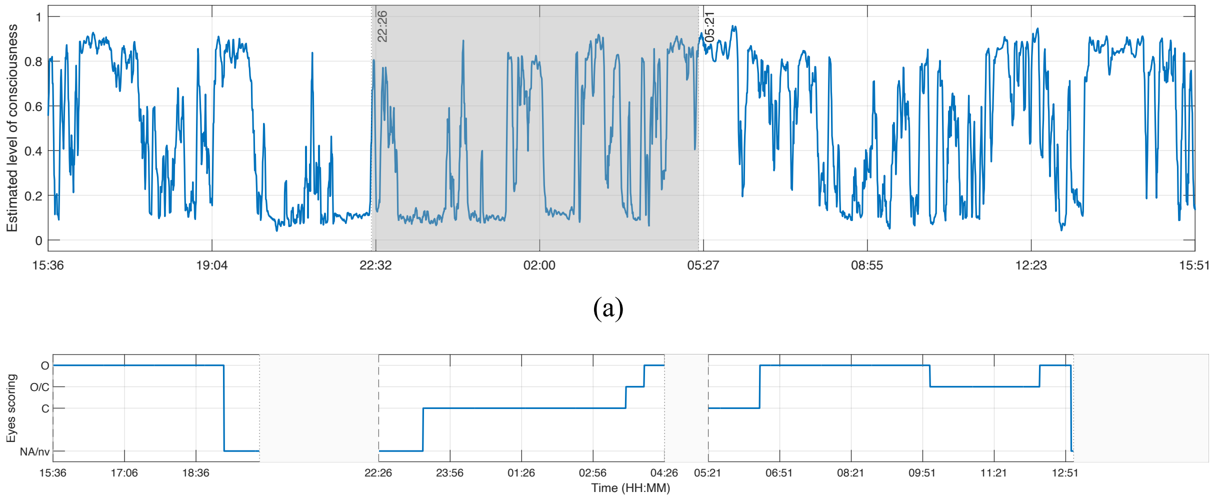


(a)

(b)

*Figure S 11: Estimated consciousness level (a) and Eyes scoring (b).*

*Table S 11: Spearman correlation coefficients between the features and the estimated levels of consciousness.*

| Features | FCM | GMM | Ensemble |
|---|---|---|---|
| $P_{theta}$ | 0,3046 | 0,3232 | 0,3032 |
| $P_{beta}$ | 0,8251 | 0,9502 | 0,8546 |
| SEF95 | 0,8565 | 0,9599 | 0,8855 |
| ERR | 0,3153 | 0,3876 | 0,3142 |
| LZC | 0,7810 | 0,8463 | 0,8053 |
| $iCOH_{theta}$ | -0,0385 | -0,0733 | -0,0447 |
| wSMI | 0,4238 | 0,3470 | 0,4008 |

## *Patient L16*

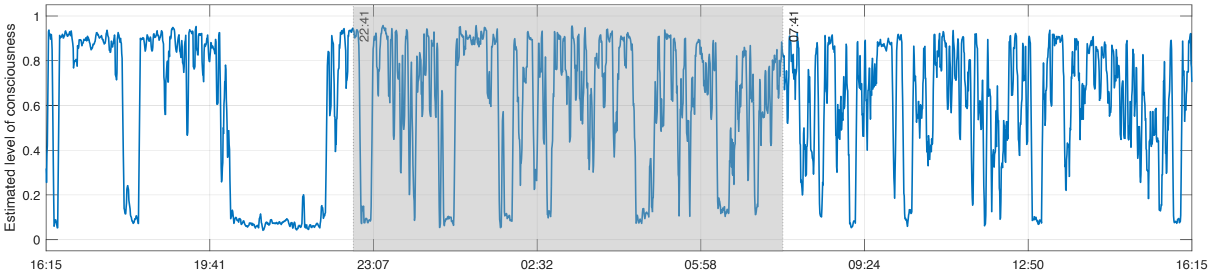


(a)

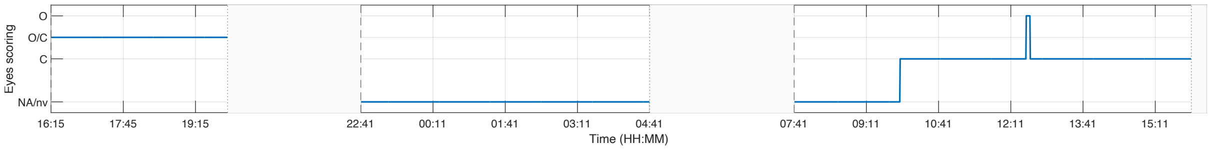


(b)

*Figure S 12: Estimated consciousness level (a) and Eyes scoring (b).*

*Table S 12: Spearman correlation coefficients between the features and the estimated levels of consciousness.*

| **Features** | **FCM** | **GMM** | **Ensemble** |
|---|---|---|---|
| $P_{theta}$ | -0,0147 | 0,0305 | -0,0143 |
| $P_{beta}$ | 0,8654 | 0,9810 | 0,8722 |
| SEF95 | 0,8661 | 0,9584 | 0,8705 |
| ERR | 0,1263 | 0,2056 | 0,1275 |
| LZC | 0,8244 | 0,9042 | 0,8294 |
| $iCOH_{theta}$ | 0,0120 | 0,0253 | 0,0119 |
| wSMI | 0,5141 | 0,4868 | 0,5077 |

### *Patient S2*

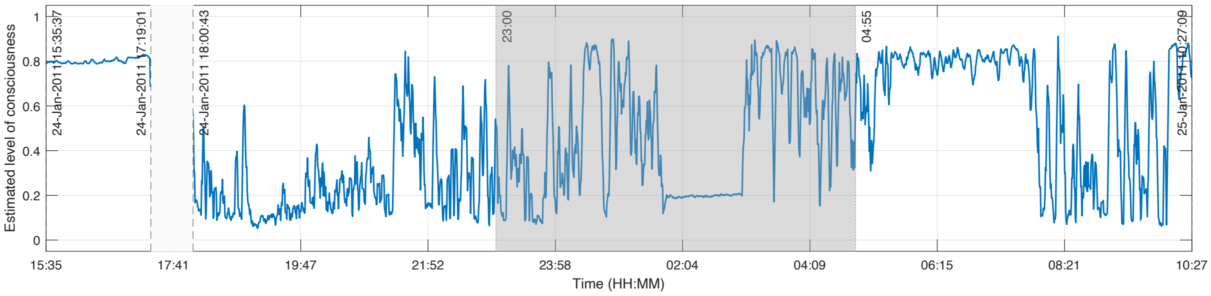


(a)

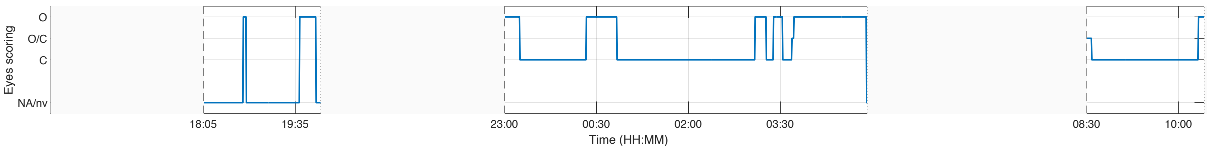


(b)

*Figure S 13: Estimated consciousness level (a) and Eyes scoring (b).*

*Table S 13: Spearman correlation coefficients between the features and the estimated levels of consciousness.*

| Features | FCM | GMM | Ensemble |
|---|---|---|---|
| $P_{theta}$ | -0,0914 | -0,4109 | -0,1643 |
| $P_{beta}$ | 0,6822 | 0,6946 | 0,7423 |
| SEF95 | 0,7918 | 0,8513 | 0,8359 |
| ERR | 0,4830 | 0,2671 | 0,4760 |
| LZC | 0,4236 | 0,3742 | 0,4545 |
| $iCOH_{theta}$ | 0,0025 | -0,0740 | -0,0088 |
| wSMI | -0,3249 | -0,5361 | -0,4002 |

## *Patient S5*

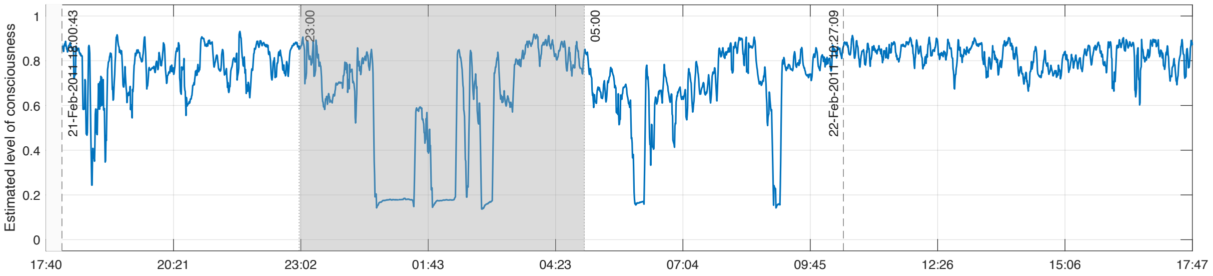


(a)

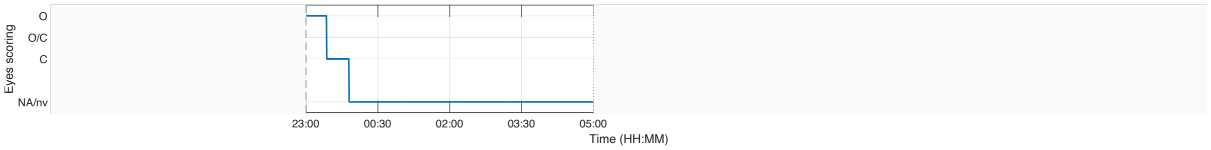


(b)

*Figure S 14: Estimated consciousness level (a) and Eyes scoring (b).*

*Table S 14: Spearman correlation coefficients between the features and the estimated levels of consciousness.*

| **Features** | **FCM** | **GMM** | **Ensemble** |
|---|---|---|---|
| $P_{theta}$ | 0,1436 | -0,5158 | 0,2113 |
| $P_{beta}$ | 0,4884 | -0,9269 | 0,5399 |
| SEF95 | 0,8262 | -0,6649 | 0,8544 |
| ERR | 0,0440 | -0,6656 | 0,1264 |
| LZC | 0,7650 | -0,1689 | 0,7277 |
| $iCOH_{theta}$ | 0,0573 | -0,0629 | 0,0631 |
| wSMI | -0,4952 | 0,3101 | -0,4814 |

## *Patient S6*

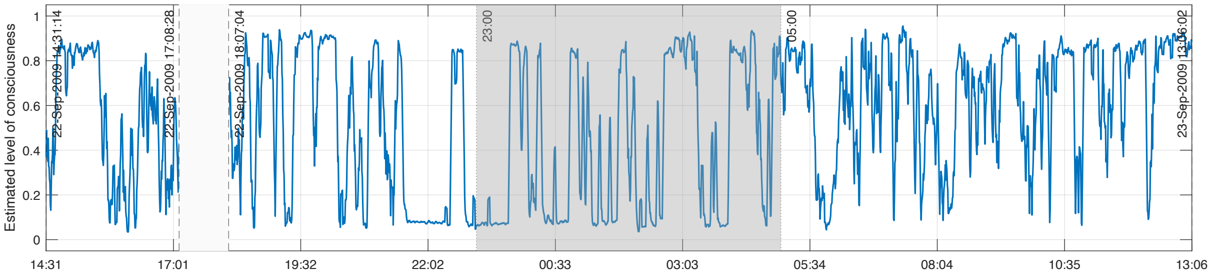


(a)

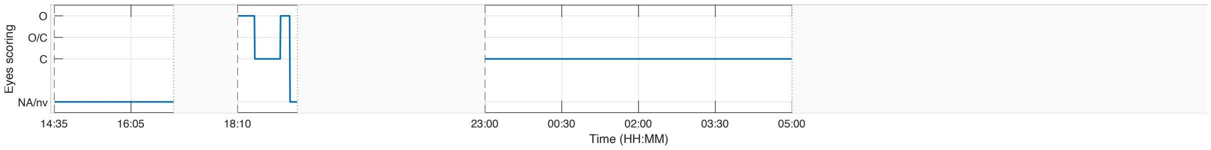


(b)

*Figure S 15: Estimated consciousness level (a) and Eyes scoring (b).*

*Table S 15: Spearman correlation coefficients between the features and the estimated levels of consciousness.*

| Features | FCM | GMM | Ensemble |
|---|---|---|---|
| $P_{theta}$ | 0,4876 | -0,4458 | 0,4740 |
| $P_{beta}$ | 0,8475 | -0,9155 | 0,8595 |
| SEF95 | 0,8241 | -0,8996 | 0,8379 |
| ERR | 0,7944 | -0,8146 | 0,7930 |
| LZC | -0,1260 | 0,0128 | -0,1033 |
| $iCOH_{theta}$ | -0,0385 | 0,0537 | -0,0458 |
| wSMI | -0,1309 | 0,1367 | -0,1457 |
